# The Role of Pericytes in Neurovascular Unit Remodeling in Brain Disorders

**DOI:** 10.3390/ijms15046453

**Published:** 2014-04-16

**Authors:** Ayman ElAli, Peter Thériault, Serge Rivest

**Affiliations:** Neuroscience Laboratory, CHU de Québec Research Center and Department of Molecular Medicine, Faculty of Medicine, Laval University, 2705 Laurier boul., Québec City, QC G1V 4G2, Canada; E-Mails: peter.theriault@crchuq.ulaval.ca (P.T.); serge.rivest@crchul.ulaval.ca (S.R.)

**Keywords:** pericytes, neurovascular unit, tissue remodeling, tissue repair, signal transduction, Alzheimer’s disease, stroke

## Abstract

Neurons are extremely vulnerable cells that tightly rely on the brain’s highly dynamic and complex vascular network that assures an accurate and adequate distribution of nutrients and oxygen. The neurovascular unit (NVU) couples neuronal activity to vascular function, controls brain homeostasis, and maintains an optimal brain microenvironment adequate for neuronal survival by adjusting blood-brain barrier (BBB) parameters based on brain needs. The NVU is a heterogeneous structure constituted by different cell types that includes pericytes. Pericytes are localized at the abluminal side of brain microvessels and contribute to NVU function. Pericytes play essential roles in the development and maturation of the neurovascular system during embryogenesis and stability during adulthood. Initially, pericytes were described as contractile cells involved in controlling neurovascular tone. However, recent reports have shown that pericytes dynamically respond to stress induced by injury upon brain diseases, by chemically and physically communicating with neighboring cells, by their immune properties and by their potential pluripotent nature within the neurovascular niche. As such, in this paper, we would like to review the role of pericytes in NVU remodeling, and their potential as targets for NVU repair strategies and consequently neuroprotection in two pathophysiologically distinct brain disorders: ischemic stroke and Alzheimer’s disease (AD).

## 1. Introduction

**Figure 1.**
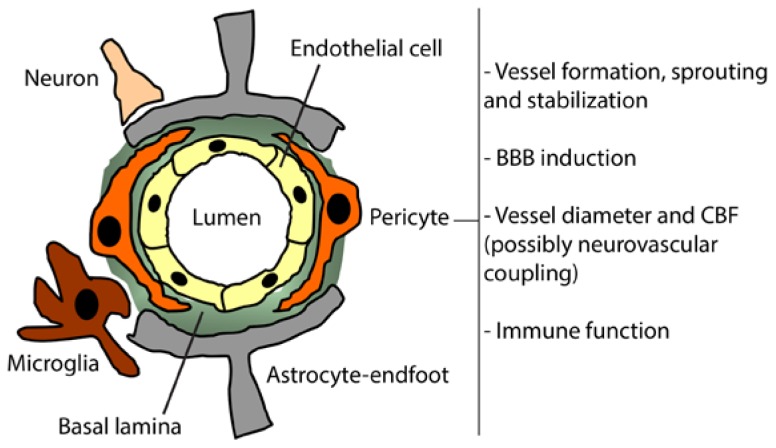
The role of pericytes at the neurovascular unit (NVU) in the healthy brain. The NVU is constituted by specialized endothelial cells, which form the blood-brain barrier (BBB), that actively interact with the basal lamina, pericytes, astrocyte-endfeet, microglia and neurons. The pericytes play an important role in maintaining NVU physiological functions by controlling tight junction (TJ) protein expression and BBB induction, microvascular stability and microvessel diameter. Pericytes might act as pluripotent cells and might have immune function at the NVU.

The brain consumes up to 20% of nutrients—mainly glucose—and oxygen present in the blood [1]. Therefore, neurons totally rely on the brain’s highly dynamic and complex vascular network that assures an accurate and adequate distribution of oxygen and glucose [2]. Therefore, optimal brain perfusion and brain vascular integrity are essential for proper brain function. For instance, a reduction in cerebral blood flow (CBF) induces a rapid neuronal stress, which can evolve to irreversible damage if this reduction persists and/or is greater than 80% [3]. The blood-brain interface, which is constituted by the blood-brain barrier (BBB), plays a central role in controlling the brain microenvironment and homeostasis [4]. The BBB is formed by tightly sealed endothelial cells, constituting a non permissive physical barrier that separates the blood from the brain. The tight junction (TJ) contacts seal endothelial cells together and induce the BBB’s polarization that is characterized by the presence of two functionally distinct sides, the luminal side facing blood circulation, and the abluminal side facing brain parenchyma [4]. Brain endothelial cells actively interact with extracellular matrix proteins forming the basal lamina, pericytes, astrocytes, microglia and neurons, forming all together the neurovascular unit (NVU) that couples neuronal activity to vascular function by controlling regional CBF (rCBF) [5], and BBB parameters [6] (Figure 1). The BBB is complemented by sophisticated transport systems, which allow continuous and precise control of brain homeostasis, among which are the ATP-Binding Cassette (ABC) transporter family, namely ABCB1 (*i.e.*, Multi Drug Resistance Protein; Mdr-1) that contributes to brain detoxification by removing toxic compounds and metabolites from the brain, among which are Aβ peptides [7]. Pericytes are required for a proper brain vascularization during the embryonic stage, mainly by stabilizing the newly formed vessels [8] and by inducing BBB functional properties during the early postnatal stage [9]. Under physiological conditions, pericytes cover around 80% of brain microvessels [10,11], outlining the importance of these cells in brain microvascular functions [12]. Several brain disorders trigger NVU remodeling, which translates the molecular and cellular responses orchestrated at the NVU upon injury. Recent reports have demonstrated that pericytes dynamically respond to stress induced by injury upon brain diseases, and actively contribute to NVU maintenance and repair upon injury. In this paper, we will briefly review the role of pericytes in brain vascular network formation, and will describe the response of pericytes to NVU injury in two brain disorders that are pathophysiologically different, which are ischemic stroke and Alzheimer’s disease (AD). Moreover, we will outline the potential of pericytes as targets for innovative therapeutic approaches that aim to restore NVU function, and consequently rescue neuronal function in these two brain disorders.

## 2. Pericytes

Pericytes were described for the first time by Rouget in 1873, and hence were named Rouget cells, but renamed afterwards by Zimmermann in 1923 “pericytes” in respect to their unique localization in the perivascular space of brain vessels (*i.e.*, surrounding (peri) brain endothelial cells (cytes)). The pericyte population in the brain is heterogeneous, and until now there has been no formal definition of these cells. Nonetheless, its is now widely accepted that mature pericytes can be defined as cells in the perivascular space embedded at the basal lamina, which are tightly associated to brain vessels through elongated processes through the basal lamina, establishing cell-to-cell contacts with endothelial cells via gap junctions [13]. Until recently [14], initial studies have suggested a contractile nature of pericytes, pointing towards their possible contribution in controlling rCBF and probably neurovascular coupling [15]. However, their precise role in controlling neurovascular tone and rCBF is still a matter of debate [16]. Although discovered 140 years ago, the scientific community only recently began to gain more insight into the function of pericytes in the brain, due to the lack of a specific and reliable marker. Investigating pericyte cell biology represents a great challenge due to the heterogeneity of these cells, and the lack of pan specific markers. As such, the strategy used now to identify these cells combines their morphology, location, and the expression of some validated markers [17]. These markers include (i) validated markers such as platelet-derived growth factor receptor β (PDGFRβ), chondroitin sulfate proteoglycan 4 (*i.e.*, neuronal/glial 2; NG2), alanyl aminopeptidase (CD13), α-smooth muscle actin (αSMA), desmin; and (ii) markers to be validated such as regulatory of G-protein signaling protein 5 (RGS5), ATP-binding cassette transporter subfamily C member 9 (ABCC9; *i.e.*, SUR2), potassium inwardly rectifying channel subfamily J member 8 (Kir6.1), endosialin, and delta-like 1 homolog (DLK1). For more details please refer to [13]. Very recently, and based on some validated markers, several transgenic mice have been developed, such as NG2dsRed [18] and EYFP-NG2 [19] mice, which would constitute great tools to better understand the role of pericytes in the brain, especially by allowing their brain intravital live imaging.

## 3. Pericyte Function in the Healthy Brain

### 3.1. Brain Vessels Sprouting

The vascularization of the brain takes place through angiogenesis that translates the formation of blood vessels via the sprouting, splitting and invasion of new endothelial tubes from existing ones [20]. More precisely, endothelial tubes sprout by invading the embryonic neural tube through the neuropil, and migrate in the direction of ventricles. Endothelial tubes develop from differentiating endothelial cell precursors, which are mesodermal-derived perineural vascular plexus precursors, called angioblasts. The angioblasts differentiate into endothelial cells that form interconnected endothelial tubes, thus forming the primitive network of brain vessels. During this process, endothelial cells secrete a wide range of bioactive molecules that specifically trigger the attraction, mobilization, and recruitment of pericytes at the abluminal side of the newly formed vascular network [21]. Brain pericytes originate from both mesoderm-derived mesenchymal stem cells and neuroectoderm-derived neural crest cells, depending on the location within the developing vascular network [20]. The recruitment of pericytes, along with the production of extracellular matrix proteins that form the basal lamina, triggers the formation of a stable and mature vascular network. Under physiological conditions, the microvasculature in the adult brain enters a quiescent stage, and pericyte migration stops. Nonetheless, under pathophysiological conditions this process is reactivated in the adult diseased brain, which will be discussed in more detail below.

### 3.2. BBB Formation and Induction

Brain microvasculature forms the BBB that restricts the movement of molecules and ions between the blood and the brain. The BBB plays a crucial role in maintaining proper neuronal function, and protects the brain from injury and disease [1]. The BBB is characterized by the presence of tight and adherens junctions between brain endothelial cells that are endowed with a high quantity of mitochondrial content, a very low rate of endocytosis, the lack of membrane fenestrations and a basal pinocytotic activity [4]. Although long speculated to play a critical role in BBB formation and induction, only recently, pericytes have been demonstrated to contribute to the formation and maintainenance of the BBB *in vivo* in both the developing and adult brain. Pericytes in the brain cover around 80% of brain microvessels [10], which is the highest rate of coverage observed among all other organs [13]. It has been suggested that this particularity is related to the central role of pericytes in maintaining BBB function [10,11]. In parallel, astrocyte-endfeet ensheath up to 90% of brain microvessels, and their role in maintaining BBB function is well established (for review see [22]). However, recent reports are suggesting that pericytes play a crucial role in inducing the physical and functional properties of the BBB, which can encompass the role of astrocytes. For instance, it has been reported that the physical properties of the BBB can still be induced in the absence of astrocytes in mice lacking functional astrocytes, the glial fibrillary acidic protein (GFAP) knockout mice [23]. Interestingly, BBB induction and maintenance in these mice were suggested to be due to increased microvascular pericyte coverage. The central role of brain pericytes in BBB function was outlined recently when their depletion in a new adult viable mouse model abolished BBB functional properties [24]. In parallel, the authors unraveled that pericytes control BBB function in at least two ways, by specifically dowregulating the endothelia genes encoding for proteins involved in endothelial cell fenestration and transendothelial permeability, namely plasmalemma vesicle-associated protein (PLVAP), and by inducing astrocyte-endfeet polarization, thus enhancing astrocyte-endfeet/endothelial contacts [24].

### 3.3. Brain Vessel Diameter and CBF Regulations

Optimal neuronal function requires an adequate cerebral blood supply. Cerebral blood supply is highly dependent on brain macro- and microvascular integrity. Brain vasculature controls cerebral blood supply through a mechanism that necessitates a high coordination among the components of the NVU, called neurovascular coupling [25]. NVU couples regional brain activity to cerebral microcirculation by adjusting local blood supply depending on brain needs [15]. In the adult brain, neurovascular coupling translates the spatial and temporal changes of CBF dictated by the metabolic status of neurons and neuronal activity. For instance, rCBF increases in response to the increased metabolic demand generated by neurons [25]. Due to their contractile nature, pericytes have been suggested to contribute to rCBF [14]. However, the ability of pericytes to regulate CBF has been a matter of debate, and is still not fully elucidated [16]. Therefore, in order to remediate the controversial reports investigating their contractile nature and their contribution in controlling rCBF, it has been suggested that pericytes may not be all contractile, and only a subset or subsets of these cells have this capacity. Ultrastructural and immunohistochemical studies have firstly suggested that pericytes are contractile cells involved in the regulation of rCBF in response to biological mediators and neural activity [26]. These studies showed that brain pericytes express several contraction-related proteins, namely alpha-smooth muscle-specific isoform of actin (α-SMA), tropomyosin and desmin [27]. In addition, functional studies showed that pericyte contraction or relaxation can be triggered by several vasoconstrictor or vasodilator mediators that activate the respective receptors and ion channels expressed in these cells. Moreover, pericytes have been reported to dilate in response to protons or adenosine, which are indicators of an increased metabolic demand [28], supporting the potential role of pericytes in coupling rCBF with the metabolic demand of neighboring neurons. Furthermore, a recent *in vivo* study further outlined the possible contribution of brain pericytes in controlling rCBF [14]. More precisely, it has been demonstrated that pericyte contraction could be induced by the administration of a vasoactive mediator, such as thromboxane agonist (U46619), in an intact adult mouse brain, triggering brain microvessels constriction, which was accompanied by an important decrease in rCBF [14]. In addition, in an elegant study, it was demonstrated that the electrical stimulation of retinal pericytes evoked localized microvessel constrictions, which propagated extremely fast to constrict distant pericytes, outlining the dynamic communication among these cells [29]. In parallel, the authors showed that superfused adenosine triphosphate (ATP) in retina or noradrenaline in cerebellum resulted in the constriction of microvessels by pericytes and glutamate administration reversed the constriction of pericytes induced by noradrenaline. These results outline the possible role of pericytes as modulators of CBF in response to changes in neural activity [29].

### 3.4. Immune Function

The immunoactive properties of pericytes are still a matter of debate. However, several recent reports are suggesting that pericytes actively contribute to the immune responses at the NVU, especially by integrating signals from the periphery and the brain [4], thus contributing to brain homeostasis due to their microvasculature proximity. Recent studies have demonstrated that pericytes are implicated in brain immune responses at different levels including (a) secretion of immune-active molecules; (b) macrophage-like activity; (c) antigen presentation; and (d) regulation of leukocyte trafficking to the inflammation sites. Brain pericytes have been shown to act as immune cells by expressing receptors of the innate immune system (e.g., pattern recognition receptors or PRRs) including toll-like receptor 4 (TLR4) and by responding to microenvironmental cues [30]. An *in vitro* study using primary mouse brain capillary pericytes revealed that pericytes express at basal levels several interleukins (IL) namely IL9, IL10, IL12 (p70), IL13, IL17 and other cytokines and chemokines including tumor necrosis factor-alpha (TNFα), interferon-gamma (IFNγ), granulocyte-macrophage colony stimulating factor (GM-CSF), granulocyte-colony stimulating factor (G-CSF), eotaxin, CCL3 and CCL4 [31]. Under inflammatory conditions, pericytes respond to the immune challenge by increasing the expression of typical inflammatory molecules, such as reactive oxygen species (ROS), nitric oxide (NO), IL1β, IL6, TNFα and matrix metalloproteinases (e.g., MMP2 and MMP9), which, in parallel, contribute to pericyte detachment and migration [31,32]. Moreover, it has been reported that the stimulation of primary porcine brain microvessel pericytes with TNFα, IL1β, IFNγ or lipopolysaccharide (LPS) increases the expression of inducible nitric oxide synthase (iNOS) and cyclooxygenase 2 (COX2) [32]. Interestingly, pericyte stimulation by LPS increases the expression of IL12, IL13 and IL9, which have been shown to be implicated in NVU signaling [31]. In addition, it has been shown that pericytes produce transforming growth factor-beta (TGFβ) that also acts as an important signaling molecule and immunoregulatory molecule at the BBB [33]. Pericytes can be activated via TLR4 triggering the expression of several macrophage markers including CD11b (integrin αM), ED2, Fc receptors (FcR) and scavenger receptors (SR) [34–36]. It has been reported that pericytes have numerous lysosomes that express acid phosphatases implicated in phagocytic activity [34]. In parallel, these reports suggested that brain pericytes may behave like macrophages in the brain [37], due to their capability to internalize small soluble molecules originating from the blood circulation and the parenchyma through interstitial fluid by pinocytosis, phagocytosis and receptor-mediated endocytosis [38]. It is important to mention that the above mentioned studies were mostly ultrastructural studies, which still need to be confirmed using more advanced technical approaches. Interestingly, *in vitro* brain pericytes express at basal levels some adhesion molecules namely intercellular adhesion molecule 1 (ICAM1) and vascular cell adhesion molecule 1 (VCAM1), which are implicated in leukocyte recruitment [39] and cellular adhesion in a major histocompatibility complex (MHC) class II-dependent antigen presentation manner [40]. In addition, an *in vivo* study using a pericyte-deficient mouse model (*pdgfrβ*^−^*^/^*^−^) revealed that ICAM1 was significantly upregulated in brain vasculature, which was accompanied by an increased number of infiltrating leukocytes into some region of the brain, thus suggesting an important role of pericytes in NVU immune quiescence that limits leukocytes brain infiltration [9]. Moreover, the stimulation of pericytes *in vitro* by TNFα has been shown to increase the production of chemo-attractant molecules, consequently increasing the expression of adhesion molecules such as ICAM1 and VCAM1 in brain microvessels, which was associated with a decrease in TJ expression, thus promoting the recruitment and transmigration of monocytes and lymphocytes into the brain [39]. In this context, pericytes can de-differentiate into antigen presenting cells and may initiate a local pro-inflammatory response. More precisely, brain rat pericytes respond to IFNγ by increasing the expression of MHC class II and antigen presentation to primed lymphocytes [39]. Interestingly, recent studies revealed that pericytes regulate leukocyte trafficking into sites of inflammation in addition to promoting their recruitment in the brain [41,42]. A confocal intravital microscopy study demonstrated that pericytes facilitate neutrophil subendothelial cells migration to inflamed sites in an ICAM1, lymphocyte function-associated antigen 1 (LFA1) and macrophage 1 antigen (Mac1)-dependent manner [41]. In addition, NG2+/α-SMA+ subset of pericytes found along capillaries and arterioles, are able to sense danger-associated molecular patterns (DAMPs), triggering a pro-inflammatory phenotype of pericytes, which was associated with an increased production of macrophage migration inhibitory factor (MIF) by these cells [42]. MIF production by activated NG2+ pericytes triggers the chemotactic migration of interstitial leukocytes and promotes their survival [42]. Moreover, leukocyte’s ICAM1-dependent interaction with TNF-stimulated pericytes triggers the upregulation of TLRs, integrins and MMPs on neutrophils and monocytes, which suggest a potential mechanism implicated in their higher motility observed *in vivo* [42].

### 3.5. Pluripotent Cells

The pluripotent nature of pericytes has been proposed for a long time, but more work is still needed to confirm this. However, several emerging reports are shedding light on their potential role as pluripotent cells. It has been shown that pericytes isolated from adult rat brain microvessels underwent a self renewal in culture, and differentiated into cells of neuronal and glial lineages only after stimulation with neuronal inducers, forming adherent clusters and non adherent spheres [43]. In the absence of neuronal inducers the primary pericytes maintained their properties and did not differentiate into other cell lineages. Finally, it has been reported that brain microvessels, isolated from transgenic mice (Immortomouse^®^, Charles River Laboratories, Wilmington, MA, USA), in culture stimulated with neuronal inducers gave rise to neurospheres and clusters, which originated from pericytes present at the basal lamina of brain microvessels. The formation of spheres and clusters in brain microvessels culture was faster than primary pericytes culture, which was attributed to neuronal inducers produced by endothelial cells in brain microvessels [44]. Taken together, these reports show that pericytes might act as pluripotent cells within the neurovascular niches, and have the capability to differentiate along multiple brain cell lineages depending on upstream cues. Interestingly, it has been reported that adult brain pericytes can be reprogrammed into functional induced-neuronal cells, which were able to integrate into the pre-existing neuronal network [45], outlining again the potential of these cells in acting as pluripotent cells. In this study, the authors found that pericytes isolated from the adult human cerebral cortex were reprogrammed into neuronal cells by the retrovirus-mediated co-expression of two transcription factors, sex determining region Y-box 2 (Sox2) and mammalian achaete-scute homolog 1 (Mash1) [45]. Moreover, the authors showed that reprogrammed pericytes acquired the ability of repetitive action potential firing and served as synaptic targets for other neurons, thus indicating their capability of integrating pre-existing neural networks. The authors also confirmed the pericytic origin of the neuronal reprogrammed cells *in vivo* by using the genetic fate-mapping approach in transgenic mice, where β-galactosidase expression was confined to PDGFRβ+/NG2+ cells at the microvessels of the cerebral cortex of young adult mice and was used as a cell tracer [45]. Finally, it has been reported that adult bone marrow constitutes a reservoir of pericytes and pericyte-like cells with pluripotent characteristics [46]. In the adult, mesenchymal stem cells (MSCs) are the postnatal progenitors that derive from mesoderm. MSCs could be isolated from the bone marrow, and from other tissues such as umbilical cord, muscle, dental pulp, and adipose tissue. Recently, it has been reported that a subset of pericytes (PDGFRβ+/Ki67+) that is located mainly at microvessel branching has mesenchymal characteristics and expresses several mesenchymal markers (CD105 and CD13) [47]. These cells can differentiate into mesodermal lineages and have the capacity to generate tissue-specific cell types. This subset of pericytes expresses mesenchymal markers *in vivo* and *in vitro*, but do not express glial, neuronal progenitor, hematopoietic, endothelial or microglial markers in their native state [47]. Interestingly, these pericytes showed multilineage potential towards mesodermal and neuroectodermal phenotypes, confirming their pluripotent potential [47].

## 4. Pericyte Interactions at the Neurovascular Unit

### 4.1. Cell-to-Cell Interactions

**Figure 2.**
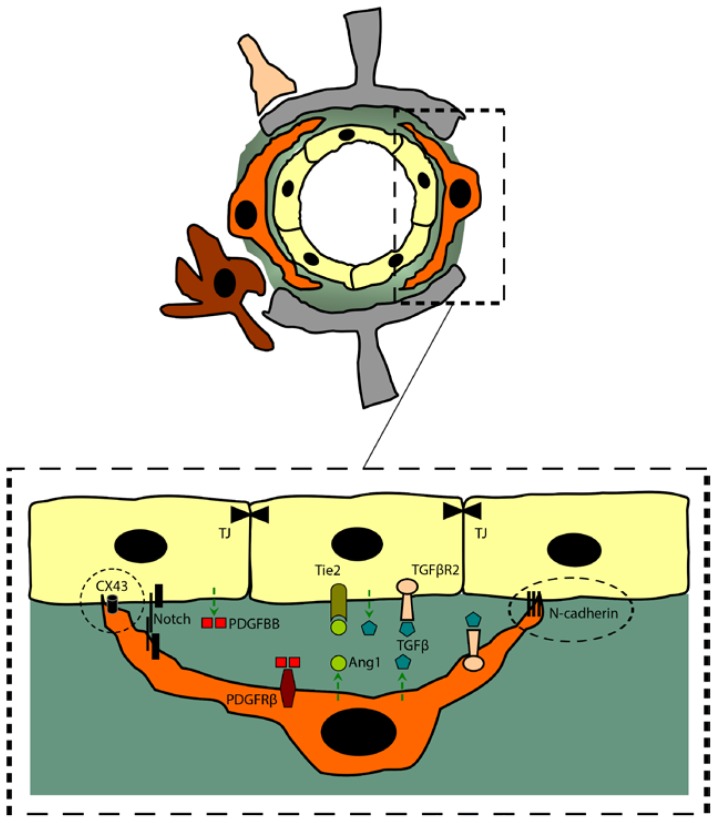
Pericyte physical and biochemical interactions at the NVU. Pericytes are embedded within the basal lamina structure and project elongated processes that wrap endothelial cells, thus establishing specialized cell-cell contacts. These contacts are mainly from peg-socket types, where cell-cell contacts are established when pericyte cell membrane protrusion-like structures (pegs) are inserted into endothelial cell membrane invaginations (pockets) cells (outlined by dashed circles). The peg-socket contacts contain cell-to-cell junction proteins, such as N-cadherin and CX43 hemichannels. CX43 hemichannels form gap junctions that allow the biochemical exchange between pericytes and endothelial cells. In parallel, several autocrine and paracrine signaling pathways govern the interaction between pericytes and endothelial cells, such as the PDGFBB/PDGFRβ signaling pathway (paracrine pathway: PDGFBB secreted by endothelial cells binds to PDGFRβ expressed on pericytes), the TGFβ signaling pathway (paracrine and autocrine pathway: TGFβ secreted by endothelial cells and pericytes binds to TGFβR2 expressed on both cell types), the Ang1/Tie2 signaling pathway (paracrine pathway: Ang1 is secreted by pericytes binds to Tie2 expressed on endothelial cells), and the Notch signaling pathway (cleavage-induced signaling pathway: the sequential proteolytic cleavage and release in the intracellular space of the Notch intracellular domain (NICD) that translocates to the nucleus and controls downstream gene expression).

Pericytes express several sets of integrins, through which they are attached to extracellular matrix proteins of the basal lamina and endothelial cells [13]. Pericytes project elongated processes that ensheath endothelial cells, establishing with the latter specialized cell–cell contacts. Although embedded at the abluminal side of brain endothelial cells, ultrastructural studies have revealed that the basal lamina almost continuously separates the pericytes from adjacent endothelial cells, where several types of cell–cell contacts have been described [13]. In the areas where basal lamina fully separates pericytes from endothelial cells, cell–cell contacts are established via adhesion plaques, which are constituted of fibronectin-rich compact monofilament bundles present between the cell membrane of pericytes and the adjacent endothelial cell membrane. However, at some sites the basal lamina contains hole-like structures, through which both cells establish direct physical contacts. These contacts are mainly from the peg-socket types, where cell-cell contacts are established when pericyte cell membrane protrusion-like structures (pegs) are inserted into endothelial cell membrane invaginations (pockets) [13], thus anchoring both cell types together. The peg-socket contacts contain cell-to-cell junction proteins, namely N-cadherin and connexin 43 (CX43) hemichannels. CX43 hemichannels form gap junctions allowing the transfer and exchange of nutrients, metabolites, secondary messengers and ions between the two cell types [48,49] (Figure 2).

### 4.2. Signaling Pathways

Due to their spatial localization within the NVU, pericytes communicate with their microenvironment via several paracrine and autocrine signaling pathways. Among these signaling pathways, PDGFRβ, transforming growth factor-beta (TGFβ), angiopoietin 1 (Ang1)/Tie2 and Notch pathways play major roles in controlling NVU establishment, maintenance and stability [20] (Figure 2).

(a) PDGFBB/PDGFRβ: The platelet-derived growth factor B (PDGFB) signaling through PDGFRβ plays a crucial role in endothelial-to-pericyte interaction, specifically in the recruitment of brain pericytes into the abluminal side of endothelial cells [50]. In angiogenic context, endothelial cells secrete PDGFB in an active homodimer form (PDGFBB), promoting the proliferation and migration of PDGFRβ-expressing pericytes within the angiogenic endothelial sprouts [51]. PDGFRβ is a tyrosine kinase receptor that is expressed on developing and mature pericytes [11]. PDGFBB binding to PDGFRβ induces the latter’s dimerization, autophosphorylation, activating several downstream signaling cascades that include survival pathways [20,51]. Moreover, it has been shown that the recruitment and attachment of pericytes to the abluminal side of the nascent blood vessels requires the interaction between the *C*-terminal retention motif of the PDGFB and the heparan sulfate proteoglycans contained within the basal lamina, thus activating PDGFRβ signal transduction [20,50,52]. More importantly, it has been demonstrated that the complete deletion of PDGFB or PDGFRβ in transgenic mice results in a perinatal lethality that was partly caused by a pronounced vascular leakage due to a mural cells deficiency [8,53]. It is noteworthy here to mention that pericytic PDGFRβ signaling seems to play an important role, not only in the brain microvasculature, but also in coronary microvasculature, as the blockade of PDGFRβ signaling with tyrosine kinase inhibitor such as Sunitinib lead to pericyte loss [54], outlining the importance of this pathway in pericyte survival and function in other vascular systems.
(b) TGFβ: TGFβ has a pivotal role in vascular development including the induction of pericytes differentiation and adhesion to brain microvessels, and the regulation of endothelial cell proliferation and differentiation [13,50]. Endothelial cells, neurons, glial cells and pericytes secrete the latent form of TGFβ, which is activated by thrombospondin or integrins [13,50]. In both pericytes and endothelial cells, activated TGFβ can bind to TGFβ receptor type II (TGFβR2) leading to the recruitment and the activation of the TGFβ receptor type I (TGFβR1) and activin-like kinase 1 or 5 (ALK1/5), thus inducing the activation and the nuclear translocation of Smad proteins that promotes transcriptional changes [13,55]. It has been shown that binding of endothelially secreted TGFβ to pericytic TGFβR2 inhibits their proliferation while inducing the expression of contractile proteins and promoting the production of extracellular matrix proteins [56]. In fact, genetic deletions within TGFβ signaling pathway components lead to a faulty vascular development resulting in embryonic lethality [13,50]. More precisely, specific knockout of the *Smad4* gene in the brain endothelium leads to several vascular defects, such as pericyte detachment and reduced capillary coverage, increased endothelial cell proliferation, vasodilatation and intraventricular hemorrhage during the perinatal period [57]. Interestingly, these results were associated with a reduction in N-cadherin expression, which is an important adhesion molecule implicated in endothelium-pericyte interaction.
(c) Ang1/Tie2: Ang1 has been shown to be predominantly expressed in perivascular mesenchymal cells, including pericytes [50]. On the other hand, the Ang1 receptor, Tie2, has been shown to be predominantly expressed on endothelial cells [50]. As such, Ang1/Tie2 signaling pathway forms a paracrine loop that has inverted orientation in comparison with PDGFB/PDGFRβ. The Ang1/Tie2 signaling pathway has been reported to play an important role in inducing endothelial cell maturation and stability, thus decreasing vascular permeability [50,58].
(d) Notch: The role of Notch signaling is well defined in neurovascular development. Establishing a cell-to-cell contact is a prerequisite for an efficient Notch signal transduction, as Notch forms heterodimeric transmembrane receptors with the transmembrane ligands delta-like (DLL) and jagged (JAG) on neighboring cells. Signal transduction is induced when the receptors and ligands bind, thus triggering the sequential proteolytic cleavage and release in the intracellular space the Notch intracellular domain (NICD), which translocates to the nucleus and binds the transcription factor recombination signal binding protein Jκ (RBPJκ), leading to downstream transcriptional changes [59]. More recently, it has been suggested that Notch signaling contribute to pericyte attachment and alignment at the abluminal side of brain endothelial cells, thus enhancing endothelial cell survival [60]. Moreover, Notch signaling has been demonstrated to play an important role in the regulation of PDGFRβ in vascular smooth muscle cell (VSMC), and probably pericytes [61]. However, more work is still required to fully address and decipher Notch signaling pathway at the NVU.

## 5. Pericytes in Brain Diseases

### 5.1. Ischemic Stroke

**Figure 3.**
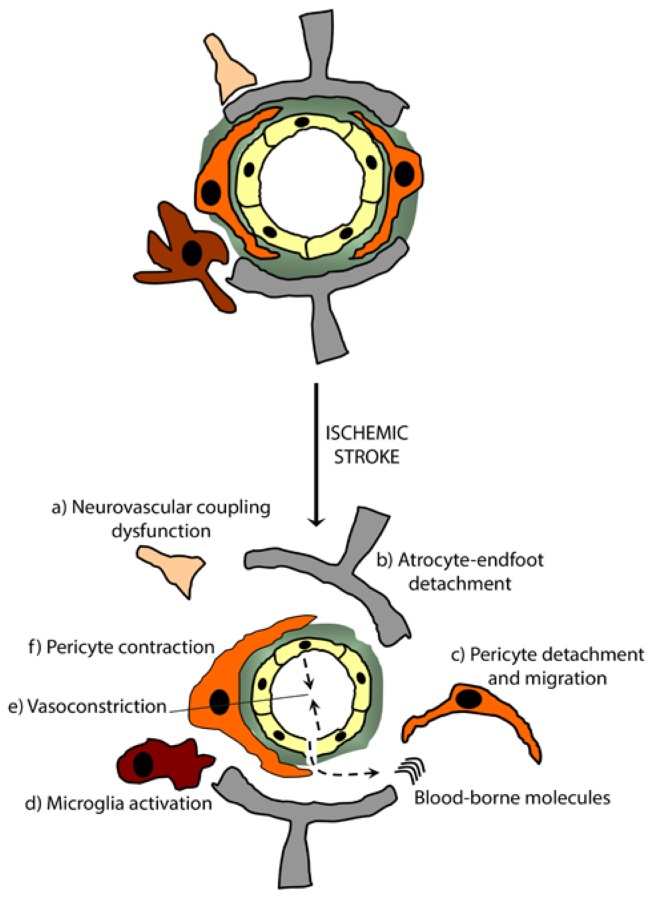
Pericyte responses upon NVU remodeling in ischemic stroke. Upon stroke, the ischemic cascade induces NVU remodeling that causes its loss of function, thus leading to the accumulation of blood-borne molecules into the brain. This loss of function is translated by (a) an impaired rCBF; (b) astrocyte-endfeet detachment; (c) pericyte detachment and migration; (d) microglia activation; (e) vasoconstriction and (f) pericyte contraction. Targeting NVU remodeling, in order to repair, stabilize and restitute the function of the NVU, constitutes a novel approach in developing successful strategies for treating ischemic stroke. This could be achieved by enhancing pericyte survival and in parallel by decreasing their contractility.

Acute ischemic stroke is caused by the sudden occlusion of a cerebral blood vessel, interrupting cerebral microcirculation in a specific region of the brain, thus initiating the ischemic cascade that leads to neuronal death [62]. During the ischemic cascade, the brain is not able to switch to anaerobic metabolism, and as it does not have any stored energy sources, the levels of ATP drop rapidly. Acute ischemic stroke triggers a rapid NVU injury and impairs the neurovascular coupling, leading to BBB breakdown, extracellular matrix proteins degradation, pericytes and astrocyte-endfeet detachment, and microglial cells uncontrolled activation (Figure 3). These events cause the secondary brain injury progression by abolishing the protective role of the BBB via TJ disruption, thus increasing cerebral edema, triggering the unspecific infiltration of peripheral immune cells into the brain, deregulating the immune responses within the infracted region, and increasing the inflammatory stress [63]. Tissue damage associated with ischemic stroke is heterogeneous [62], therefore it might be speculated that pericyte injury is tightly associated to this heterogeneity and that pericytes could be affected by ischemic stroke stressors in different ways ranging from slight dysfunction to death. Ischemic stroke triggers calcium (Ca^2+^) flow into cells in the damaged area. More precisely, it has been shown that ROS induces a sustained increase in intracellular Ca^2+^ of human brain pericytes in culture, leading to their contraction [64] outlining the potential role of Ca^2+^ signaling during the ischemic cascade in pericyte contraction, and impaired brain perfusion after stroke. Importantly, a recent report demonstrated that ischemic stroke induces pericytes contraction on microvessels and remained contracted despite complete reopening of the occluded artery. This no-reflow phenomenon translates the post-ischemic cerebral hemodynamic changes that deeply affect cerebral perfusion, even after obstructed vessel recanalization, which can have a deleterious effect on ischemic regional reperfusion, thus aggravating the ischemic damage. Indeed, pericyte contraction induced by the oxidative stress during the ischemic cascade triggers microvessel constrictions, therefore entrapping erythrocytes present in the blood within NVU lumen [27], and promoting the aggregation of these cells with fibrin [65]. Interestingly, these events occur while plasma flow remains uninterrupted, making it difficult to properly investigate blood reflow [27]. Moreover, ischemic stroke induces a rapid loss of pericytes at the microvessels of ischemic brain, which was reported to occur via caspase-3 activation. This loss is accompanied by the proliferation and the deposition within the basal lamina of a subset of pericytes that are PDGFRβ+, which have been shown to originate from the NVU niche of the ischemic brain [66]. These proliferative migrating cells formed a fibrotic, contracted and macrophage-laden lesion core that is somehow distinguishable from the hypertrophic astroglia rim observed usually in both experimental and human stroke [66]. These observations go along previous reports showing that up to 40% of pericytes migrated from their microvascular location at the site of impact upon traumatic brain injury [67]. The migration of pericytes resulted in thinning of the basal lamina, and the migrated pericytes appeared viable and remained in a perivascular location in the adjacent neuropil. Interestingly, the portion of pericytes that remained in place showed cytoplasmic alterations and nuclear chromatin changes, leading to their degeneration [67]. As mentioned before, pericytes play a crucial role in angiogenesis and microvessel formation and stabilization. However, this remodeling process is a double-edged sword, as the newly formed vessels are not stable, which could have a deleterious effect by increasing the risk of hemorrhagic transformation [68]. It is important to outline here that ischemic stroke triggers NVU remodeling by inducing the rapid activation of several signaling pathways involved in angiogenesis, which translates to intrinsic attempts from the brain to enhance the vascularization of the damaged region in order to increase blood supply, re-establish rCBF, enhance oxygen and nutrient import and remove toxic metabolites generated by the oxidative stress [65,68]. For instance, minutes after ischemic stroke, the genes related to angiogenesis are upregulated within the NVU. More precisely, ischemic stroke induces a rapid vascular endothelial growth factor (VEGF) expression in the brain lasting for days in neurons and up to a week in brain vasculature [69]. In parallel, ischemic stroke has been reported to increase PDGFRβ expression in pericytes present in the peri-infarct region, which was accompanied by an increased expression of PDGFB in endothelial cells in the same region. This coordinated regulation of PDGFB leads to the activation of PDGFRβ at pericytes in the peri-infarct region, thus enhancing pericyte survival [70]. Further, the Ang1/Tie2 signaling pathway has been shown to be triggered by ischemic stroke stressors contributing to the post-stroke angiogenesis [71] and this pathway is well known to promote vascular remodeling and stabilization [72]. More precisely, it has been shown that pericytic Ang1 increases the gene and protein expression of TJ proteins in brain endothelial cells via Tie2 receptor activation [73]. In parallel, ischemic stroke induces γ-secretase-dependant cleavage of Notch protein, leading to the intracellular accumulation of NICD that triggers caspase-3 cleavage and activation, and ICAM1 expression [74]. In adult brain, Notch 3 expression is enriched in pericytes and plays a crucial role in vascular stability and pericyte coverage [75]. The importance of the Notch signaling pathway at the NVU upon ischemic stroke was further demonstrated when it was reported that *Notch 3*^−^*^/^*^−^ mice were more susceptible to ischemic stroke and presented larger infracts, due to severe CBF deficits [76]. Taken together, these reports point towards a complex coordination among these signaling pathways during NVU remodeling, thus constituting interesting targets for novel approaches in ischemic stroke treatment and management.

### 5.2. Alzheimer’s Disease (AD)

**Figure 4.**
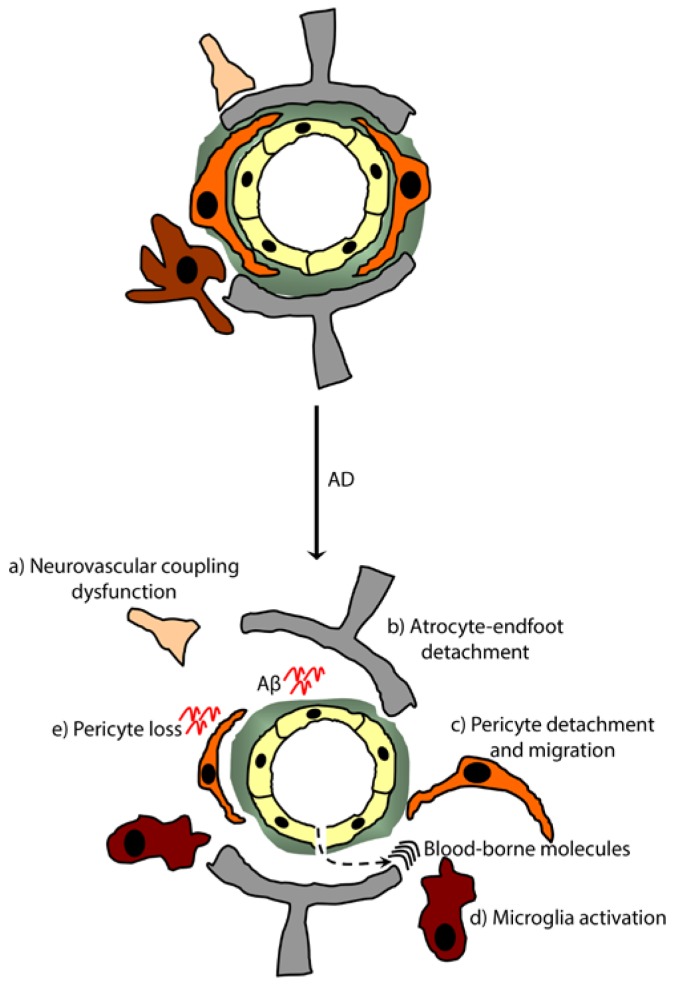
Pericyte responses upon NVU remodeling in Alzheimer’s disease (AD). In AD, the neurodegenerative cascade has been shown to be initiated by NVU remodeling that triggers its loss of function, leading to the accumulation of blood-borne molecules into the brain. This loss of function is translated by (a) rCBF dysfunction; (b) astrocyte-endfeet detachment; (c) pericyte detachment and migration; (d) microglia activation and (e) pericyte loss. Targeting NVU remodeling in order to repair, stabilize and restitute the function of the NVU, would constitute a novel approach in developing successful strategies for treating AD.

AD is the most common neurodegenerative disorder that is mainly characterized by the accumulation of the neurotoxic amyloid-beta peptide (Aβ) on blood vessels and in brain parenchyma [1]. Although neuroinflammation is widely accepted as an important phenomenon occurring in AD, accumulating evidence has pointed towards an important contribution of cerebrovascular dysfunctions in AD pathogenesis [1,77]. In fact, several AD pathological studies revealed that brain microvessels develop basement membrane thickening, endothelial cell shape changing and pericyte degeneration [78–80]. Moreover, it has been proposed that BBB leakage and altered transport, associated with hypoperfusion/hypoxia are key cerebrovascular dysfunction pathways associated with AD pathobiology [1]. As mentioned above, pericyte interactions with endothelial cells are essential to maintain the BBB’s physical and functional properties, which are essential for maintaining an optimal microenvironment suitable for neuronal function, therefore suggesting that the early loss of pericytes at the NVU contributes to AD pathogenesis [1]. BBB breakdown has been reported in AD; the disruption of tight and adherens junctions leads to an increase in endothelial transcytosis at the early stages that evolves to a paracellular permeability accompanied by the enzymatic degradation of the basal lamina, thus exacerbating the accumulation of various blood-borne molecules inside the brain (Figure 4) [1]. An *in vivo* study using a PDGFB retention motif knockout mouse model (*pdgfb*^ret/ret^) demonstrated that pericyte deficiency increases BBB permeability by specifically enhancing transendothelial transport, without affecting TJ proteins expression and paracellular transport [24]. Moreover, considering their ability to degrade various circulating proteins in their lysosomes, including immunoglobulins and fibrin, pericyte loss has been shown to exacerbate BBB breakdown [81,82]. In this regard, it was shown in pericyte-deficient mice that age-dependent loss of brain pericytes triggers a reduction in cerebral microcirculation and BBB breakdown, thus leading to neurodegeneration and cognitive impairments [83]. The authors concluded that the vascular damage observed in pericyte-deficient mice precedes neuronal damage and neuroinflammation, which suggests that primary vascular lesions can lead to neurodegeneration [83]. As discussed above, pericytes have been suggested to contribute to rCBF, and possibly neurovascular coupling [29], a role that needs to be clarified. Interestingly, impaired CBF has been observed in elderly persons before presenting AD-associated pathological symptoms, namely cognitive impairment, brain atrophy and Aβ accumulation [2,84], which suggest that moderate to severe hypoperfusion could contribute to the onset of AD pathogenesis [85]. However, the exact role of pericytes in AD-associated cerebrovascular dysfunctions, including focally reduced microvessel density and hypoperfusion, is still not fully understood [10,86]. In addition to neuroinflammation, Aβ accumulation around brain capillaries induces toxicity to pericytes [87]. It has been shown that Aβ deposition promotes an overproduction of ROS in pericytes [88], thus contributing to oxidative stress involved in BBB breakdown and loss of function. Under inflammatory conditions, pericytes express the low density lipoprotein receptor-related protein 1 (LRP1), which is a transmembrane protein involved in Aβ processing and clearance through the BBB [89], thus suggesting an important role of pericytes in Aβ clearance [31]. Using primary brain pericyte culture *in vitro*, it has been reported that these cells are able to internalize Aβ in an LRP1-dependent manner, but that their exposure to high concentrations of various Aβ species can trigger their death [87,90]. Interestingly, pericytes with an apolipoprotein E2 (apoE2) or apoE3 genotype present an increased Aβ resistance comparatively to pericytes with the apoE4 allele [91]. More importantly, a recent study using crossed transgenic mice overexpressing the Swedish mutation of human Aβ precursor protein (APP^sw/^°) with PDGFRβ^+/−^ mice revealed that loss of pericytes accelerates AD-like pathology [81]. In fact, pericytes loss in APP^sw/^° PDGFRβ^+/−^ mice caused an accelerated vascular damage and increased Aβ40 and Aβ42 levels in the brain interstitial fluid (ISF), which triggers tau pathology, early neuronal loss and cognitive function changes [81].

## 6. Pericytes in Neurovascular Unit Repair: Therapeutic Implications and Perspectives

NVU remodeling translates the molecular and cellular responses orchestrated within the structure of the NVU upon injury, in which the luminal-to-abluminal interactions are reshaped and reorganized [4]. These responses are organized by the brain to promote NVU function by inducing the remodeling of the basal lamina, the realignment of pericytes and astrocytic endfeet along the endothelial cells, the restitution of a stable BBB, and the reestablishment of neurovascular coupling. However, these attempts are not always conclusive and successful, and can even contribute to the pathobiology of brain disorders, by exacerbating neuronal injury. Moreover, it has been demonstrated that upon injury, brain microvessels that recover do not always recuperate a fully functional state. For instance, sustained vascular dysfunctions can contribute to the no-reflow phenomenon and vascular leakage in ischemic stroke, and in exacerbating the neurodegenerative cascades observed in AD. As such, it became interesting to develop novel strategies that promote more specifically NVU function and BBB restitution upon injury in order to rescue neuronal survival. Due to their spatial distribution within brain microvessels, their broad cellular properties and their intimate physical and biochemical interactions within the structure of the NVU, the pericytes constitute an ideal target to develop novel therapeutic strategies aiming to modulate and control NVU remodeling, and ultimately restore NVU function upon injury, thus consequently improving neuroproection. For instance it has been shown that the oxidative and nitrative stress associated with ischemia/reperfusion induced pericyte contraction and consequently microvessel constrictions, therefore impairing CBF [27]. Interestingly, the administration of superoxide scavenger *N*-tert-butyl-α-phenylnitrone (PBN) and a low dose of NOS inhibitor *N* -nitro-l-arginine (L-NA), after ischemia and before reperfusion restored microvessel patency and reduced brain ischemic damage [27]. Recently, we have demonstrated that pericyte coverage of brain microvessels significantly decreased upon ischemic stroke/reperfusion, which was accompanied by a decreased expression of N-cadherin [92]. Interestingly, in the same study we found out that the intracerebroventricular (i.c.v.) injection of recombinant human VEGF enhanced pericyte survival and coverage of brain endothelial cells via mechanisms involving increased N-cadherin expression on ischemic brain microvessels [92]. This increased coverage was accompanied by an enhanced CBF in the ischemic hemisphere, thus reducing brain damage. Moreover, the administration of a synthetic liver X receptor agonist (GW3965) has been reported to increase Ang1/Tie2 signaling pathway activity in the ischemic brain, thus enhancing NVU function and consequently attenuating ischemic stroke damage [93]. In addition, it has been shown that Notch signaling pathway stabilization using the γ-secretase inhibitor (DAPT) successfully attenuated ischemic stroke damage [74]. Finally, it has been reported that GFP + bone marrow derived cells injected into mice subjected to ischemic stroke integrated both the pre-existing and newly formed microvessels [94]. In this study, it was proposed that the transplanted bone marrow-derived pericytes might contribute to NVU remodeling and repair.

Recently, it has been reported that lack of pericytes initiated the neurodegenerative cascades that were associated with microvessel dysfunction, which preceded neuronal damage and neuroinflammation in AD. More precisely, it has been shown that pericyte-deficient mice exhibit increased BBB permeability, which allowed the entry of blood-borne toxic molecules into the brain and created a local hypoperfused/hypoxic microenvironment within the NVU microenvironment [10]. As such, it is tempting to verify whether rescuing brain pericytes using strategies that involve MSCs transplantation or target pericyte survival could modulate NVU remodeling and repair, which would stop, enhance or reverse the neurodegenerative cascades observed in AD. Finally, we believe that more studies are required in order to gain more insights in the interactions of pericytes with their microenvironment and within the NVU structure in health and disease. These studies would be valuable to complete the puzzle, which will allow the development of novel therapeutic approaches based on NVU repair.
